# Feasibility of Magnetic Resonance Imaging Monitoring of Postoperative Total Knee Arthroplasty without Metal Artifacts: A Preliminary Study of a Novel Implant Model

**DOI:** 10.1155/2018/8194670

**Published:** 2018-10-23

**Authors:** Xiangchao Meng, Zhe Du, You Wang

**Affiliations:** ^1^Department of Bone and Joint Surgery, Renji Hospital, School of Medicine, Shanghai Jiao Tong University, Shanghai, China; ^2^Trauma Center, Peking University People's Hospital, Beijing, China

## Abstract

**Background:**

Although magnetic resonance imaging (MRI) can provide superior detailed images of tissues without ionizing radiation, the imaging evaluation of total knee arthroplasty (TKA) complications and posttherapy changes can be limited because of abundant artifacts on MRI scans due to metallic implants and endoprosthesis in limb salvage and fracture fixation. This study aimed to develop a novel model of TKA using a polyetheretherketone- (PEEK-) on-highly cross-linked polyethylene (HXLPE) implant and to investigate its feasibility for MRI monitoring of peri-implant bone formation, the healing process, signs of infection, and tumor recurrence after TKA.

**Methods:**

Three skeletally mature goats underwent TKA with the PEEK-on-HXLPE implant. Radiographic and MRI examinations were performed at 4 weeks postoperatively. Images were analyzed for the existence of artifact interruption and postoperative changes in the bone and peripheral soft tissue.

**Results:**

The results showed that PEEK and HXLPE were invisible, but the metal wires were clearly visualized on X-rays. On MRI scans, PEEK and HXLPE materials showed a low signal intensity, fine metal wires generated no obvious metal artifacts on MRI scans, and the marrow and soft tissue showed a continuous signal intensity without artifact interruption.

**Conclusions:**

This preliminary study introduced a novel model using PEEK-on-HXLPE knee implant for in vivo MRI monitoring of the region around the implant without metal artifacts. This novel model may be used to apply MRI to observe bone formation and the healing process around the prosthesis and the signs of infection and tumor recurrence after TKA. This model may be used to improve the diagnostic accuracy of postoperative complications of TKA clinically.

## 1. Introduction

Total knee arthroplasty (TKA) is the most effective surgical treatment for patients with advanced diseases and extensive deformation of the knee and the ultimate reconstruction method for patients after joint tumor surgery [1, 2]. However, several complications can occur after TKA, such as implant loosening, infection, implant instability, osteolysis, heterotopic ossification, extensor mechanism disruption, and fracture [3–5]. Tumor recurrence, delayed union, and infections are still grand challenges for orthopedic surgeons. In order to prevent long-term adverse events caused by these complications, early radiological diagnosis and continual monitoring are important for the timely treatment of delayed union, infection, osteolysis, and local recurrences of bone and tissue sarcomas after TKA. There are multiple measurement techniques available to diagnose and continually monitor patients, ranging from radiography and computed tomography (CT) to positron emission tomography [6, 7]. MRI is a noninvasive, ideal measurement technique that does not cause radiation injury to patients. Increasing evidence has suggested that MRI demonstrates high sensitivity for detecting osseous changes, bone formation, myelopathies, soft tissue inflammation, and neurovascular changes [8, 9]. However, imaging evaluation of these changes has been limited because of metallic implants, and endoprosthetic components can be especially problematic, causing substantial artifacts on CT and MRI scans [10]. Therefore, further research of methods for reducing artifacts on MRI scans and improving image clarity and diagnostic accuracy is required [11, 12].

Fortunately, with the rapid development of biomaterials science, polyetheretherketone (PEEK) materials with excellent mechanical properties and bioinertness, which do not demonstrate toxicity or mutagenicity, teratogenicity, and carcinogenicity, have been developed and applied clinically [13]. PEEK is also radiologically transparent without artifact interruption on MRI scans. This preliminary study aimed to develop a novel animal model of TKA using the PEEK-on-highly cross-linked polyethylene (HXLPE) implant and to investigate its feasibility for MRI monitoring of bone formation, the signs of infection, healing process, and local recurrence of osteosarcoma without metal artifacts, which may help improve diagnostic and therapeutic research of postoperative complications of TKA in the future.

## 2. Materials and Methods

### 2.1. Design and Fabrication of the Goat Prosthesis

**Figure 1:**
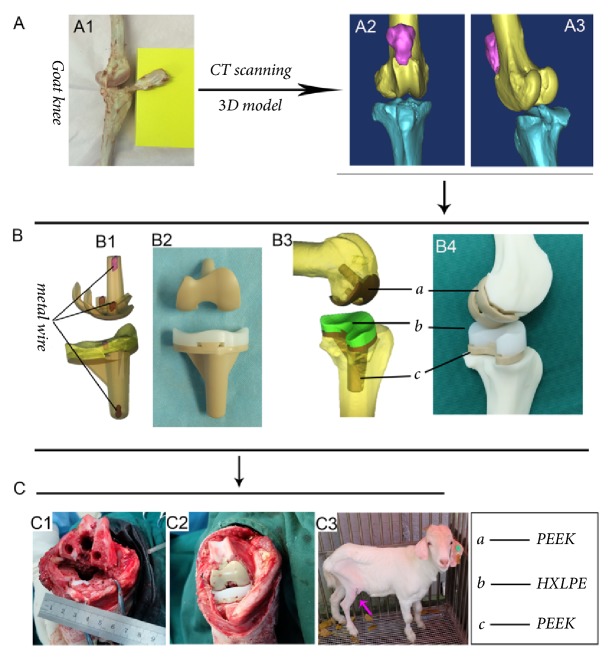
Development of a 3-dimensional (3D) model of the goat knee by computed tomography (A1-3). A 3D sketch of the polyetheretherketone- (PEEK-) on-highly cross-linked polyethylene (HXLPE) prosthesis (B1 and 3) and images of the implant (B2 and 4). The red parts in the 3D sketch (B1) represent the fine metal wires. Intraoperative images show the bone cuts and osteotomy of the distal femur and tibial plateau (C1); the prosthesis was fitted, and the bone cement was applied to the femoral and tibial sides to fix the implant (C2). The knee function of the goat is good after total knee arthroplasty (C3).

The goat prostheses and surgical instruments were designed based on CT data (Light Speed 16; GE Medical Systems, Milwaukee, WI, USA) of a standard goat's right hind limb (Figure 1(A1-3)). The PEEK component and HXLPE bearing (Zeniva PEEK ZA-500, Chirulen HXLPE 1020X) were provided by Jiangsu Okani Medical Technology Co., Ltd. (Soochow, JS, China). The prosthesis consists of a femoral component (PEEK) and a tibial tray (PEEK) with an insert bearing (HXLPE) (Figure 1(B1-3)). The tibial components include a tray and insert that lock tightly together. The prosthesis can be detected by radiography from the signals of metal wires inside the PEEK-on-HXLPE implant (Figure 1(B1)). The femoral prosthesis was sterilized by irradiation, and the tibial prosthesis was sterilized by ethylene oxide.

### 2.2. Animals

Three goats (weight, 30 kg; height, 66–70 cm) were obtained from Jiagan Biotechnology Co., Ltd. (Shanghai, China). All animal studies were performed following the appropriate guidelines (No. 2017021) and in accordance with Shanghai Jiao Tong University School of Medicine Animal Care and Use Committee Guidelines.

### 2.3. Surgical Procedures

Preoperatively, animals received intraperitoneal injections of pentobarbital sodium (Merck, Darmstadt, Germany) for general anesthesia (50 mg/kg). Surgery was performed on the right hind limb, and after the site was shaved with electric scissors and disinfected with povidone-iodine, an incision was made using the lateral parapatellar approach. Bone cuts were completed using appropriate surgical instruments, and bone cement was applied to the femoral and tibial sides to fix the implants, followed by wound irrigation with normal saline (Figures 1(C1) and 1(C2)). The muscle fascia and skin were sutured interruptedly. Antibiotics (penicillin, 60 mg/kg) were administered for 5 days postoperatively [14].

### 2.4. Postoperative Imaging

The plain lateral X-rays and MRI scans were obtained at 4 weeks postoperatively. X-rays were acquired with a commercially available digital radiography system (Definium 6000, Volume RAD, GE Healthcare), and MRI scans were acquired with a 3.0-T MRI scanner (Philips, Amsterdam, The Netherlands) using a 10-cm millipede scanner coil. Regions of interest, including the signal intensity changes of the cortical bone and medullary cavity around the PEEK material of the prosthesis and surrounding soft tissue, were analyzed on MRI by a professional radiologist.

## 3. Results

### 3.1. Radiographic Location of the PEEK-on-HXLPE Prosthesis

**Figure 2:**
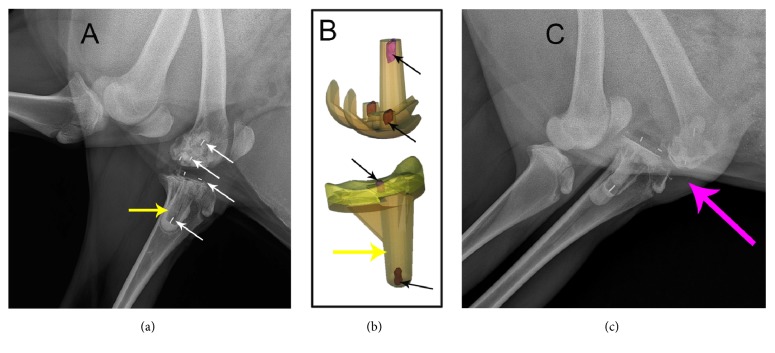
Four-week postoperative X-rays illustrating the location of the prosthesis with metal wires after total knee arthroplasty. The metal wires, shown as the high-density signal (the white arrows in (a)), were inserted in the components as the red part in the 3D sketch (the black arrows in (b)). According to the signals of the wires, the position of the tibial component, the spacer, and the femoral component can be roughly predicted. The normal prosthesis alignment position should be like the imaging in (a); however, the imaging in (c) indicated prosthesis component was dislocated or possibly got stuck. The tibia tray of PEEK prosthesis, showing low-density signal in the tibia (the yellow arrow), is cemented in the bone, and the bone cement showed slightly higher density signal than that of PEEK.

On the x-rays at 4 weeks postoperatively, the PEEK and HXLPE materials were radiologically transparent and invisible; however, fine metal wires were visualized radiographically so that the position of the prosthesis could be evaluated (Figures 2(a) and 2(b)), which might help us to locate the prosthesis intraoperatively. As shown in Figure 2(c), the wires were not in the correct position, which indicated dislocation of the joint at 4 weeks postoperatively.

### 3.2. Evaluation of the Bone and Soft Tissue Structure around the PEEK Material by MRI

**Figure 3:**
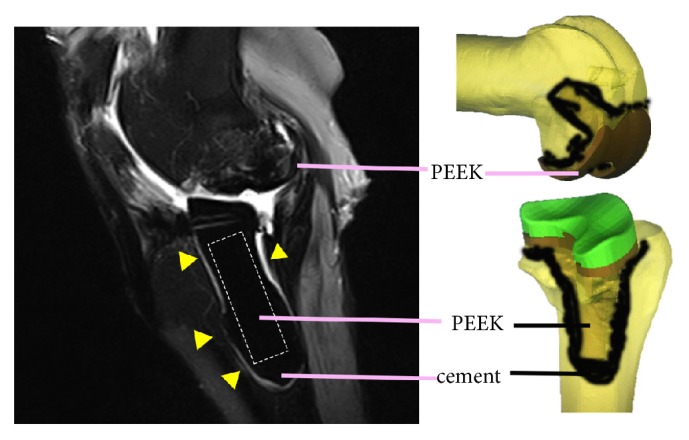
Postoperative magnetic resonance imaging illustrating the prosthesis with a low signal intensity without artifacts and marrow and soft tissue with a continuous signal intensity without artifact interruption. The hyperintense signal around the cement on the T2-weighted magnetic resonance imaging scans may represent water and proteins adsorbed on the cement surface or soft tissue edema and inflammation in the early stage of healing (the yellow triangles indicate the hyperintense signals around the cement). The fine metal wires inserted into the polyetheretherketone (PEEK) and highly cross-linked polyethylene (HXLPE) components do not produce obvious metal artifacts.

On the 4-week postoperative MRI scans, PEEK and HXLPE materials showed a low signal intensity without artifacts, and the fine metal wires did not generate obvious metal artifacts. The marrow and soft tissue showed a continuous signal intensity without artifact interruption. Increased signal intensity on the T2-weighted MRI scans was observed around the cement, which may represent water and proteins adsorbed on the cement surface or soft tissue edema and inflammation in the early stage of healing (Figure 3).

## 4. Discussion

The results of our preliminary study showed that PEEK-on-HXLPE prosthetic materials showed a low signal intensity and no obvious artifacts on MRI scans, and marrow and soft tissue showed a continuous signal intensity without artifact interruption. These results indicate that our novel model of TKA with a PEEK prosthesis may help improve diagnostic and therapeutic research of postoperative complications of TKA in the future.

MRI is an established technique for the evaluation of cancer, blood vessels, brain, and heart and articular cartilage in joint disease [15], but it is not a standard imaging modality used during joint replacement, such as TKA. Metal implants, which are now mostly used in the clinical setting, can induce inhomogeneity in the static magnetic field (B0), resulting in image distortion as metal-related artifacts, severely reducing the quality of examinations and obscuring anatomical regions, which may lead to false diagnosis or evaluation [16, 17]. However, the bone and tissue surrounding the implant can provide direct and indirect impressions of the bone structure. In addition, the key advantages of MRI are the possibilities of visualizing the neurovascular bundle and observing the inflammatory or neoplastic processes in detail. The evaluation of osseointegration around the implant with MRI may also have great potential. Thus, how can the effects of TKA metal artifacts on MRI be eliminated? Many researchers have tried to reduce or eliminate metal artifacts by adjusting the MRI scan data and switching to titanium metal, but there is no fundamental way to eliminate metal artifacts [12, 17]. Other researchers are constantly exploring biomaterials without artifacts on MRI scans to develop a new generation of TKA prosthesis to solve this problem.

PEEK is a thermoplastic polymer known to be resistant to fatigue strain and radiologically transparent. There has been growing interest in PEEK material as an arthroplasty-bearing material, such as PEEK-on-PEEK articulations in the spine as well as total knee implants [13]. Other studies reported that PEEK materials can be a superior alternative to minimizing implant artifacts on MRI or CT scans for professional use in fracture fixation, spinal surgery, dentistry, and cranioplasty. Zimel et al. [18] reported that less MRI signal loss occurred in the carbon fiber-reinforced- (CFR-) PEEK phantom than in the titanium phantom simulation, particularly as the angle increased with respect to the direction of the static magnetic field; in addition, CFR-PEEK nails had fewer MRI artifacts than titanium nails on scored T1-weighted (T1W), short inversion time inversion recovery, and contrast-enhanced T1W fat-saturation MRI sequences. Korn et al. [19] examined the suitability of MRI for assessing peri-implant bone formation exemplarily for a dental implant, PEEK coated with a thin layer of titanium, in a minipig model; they reported that MRI was promising in monitoring bone formation.

Many in vitro studies designed PEEK knee prosthesis and proved its wear-resistance [20, 21], but few studies have investigated the property of the novel prosthesis in vivo. Herein, a PEEK prosthesis was designed based on data from a 3-dimensional model of a goat's knee, and we successfully implanted it in the animal experiment. We observed the MRI images of the PEEK-on-HXLPE prosthesis in vivo and found that the prosthesis and cement showed a low signal intensity on the T2-weighted MRI scans, marrow and soft tissue showed a continuous signal intensity in T2-weighted MRI scans without artifact interruption, and tissue near the prosthesis and cement showed hyperintensity on T2-weighted MRI scans, which may indicate soft tissue edema and inflammation in the inflammatory phase and wound healing process. These results are accordance with the description of the process of osseous healing of implants on a cellular level [22]. After insertion of a biomaterial, the polymerized fibrinogen was the first tissue around the implant in the early stage of healing; water and proteins are adsorbed on the implant surface, and a coagulum is formed, occluding the vascular leak. The immune system is activated in the inflammatory phase, which is characterized by a high-water content and detectable as a hyperintense signal on T2-weighted MRI scans. During the proliferative phase, new bone formation is initiated by the deposition of osteoid matrix, which is consecutively mineralized at approximately 1 week after implant insertion [22]. Finally, the mineralized tissue, as new bone, is detectable as a region without signals, showing a black appearance on MRI scans. The great advantage of MRI is the ability to follow bone formation from early healing stages when the tissues consist of water and unmineralized structures and have no direct impressions on X-rays.

The present study examined the suitability of MRI to monitor peri-implant bone formation and tissue infection exemplarily for a PEEK-on-HXLPE knee implant in a goat model. However, there are a couple limitations to this preliminary study. First, the PEEK material is not yet prudently applied clinically as a TKA prosthesis. Further studies need to be conducted on the design and manufacturing of the PEEK prosthesis, as well as modification of the PEEK material. Second, we only observed X-ray and MRI scans at 4 weeks postoperatively, so a larger number of animals and multiple follow-up intervals should be evaluated in future experiments. Nevertheless, the result suggested this model may be used to observe the signs of early periprosthetic infections, process of bone repairing and remodeling after TKA, and features of tumor recurrence around the periprosthetic tumor, which may improve the diagnostic accuracy of related diseases.

## 5. Conclusions

This preliminary study introduced a novel model using the PEEK-on-HXLPE knee implant for in vivo MRI monitoring of the region around the implant without metal artifacts. The new biomaterial prosthesis does not produce artifacts and may allow the use of MRI to monitor bone formation, signs of infection, and tumor recurrence around the implant after TKA.

## Data Availability

The data used to support the findings of this study are included within the article.
